# Mannosylated-Chitosan-Coated Andrographolide Nanoliposomes for the Treatment of Hepatitis: In Vitro and In Vivo Evaluations

**DOI:** 10.3390/membranes13020193

**Published:** 2023-02-03

**Authors:** Sayali Pravin Metkar, Gasper Fernandes, Ajinkya Nitin Nikam, Soji Soman, Sumit Birangal, Raviraja N Seetharam, Manjunath Bandu Joshi, Srinivas Mutalik

**Affiliations:** 1 Department of Pharmaceutics, Manipal College of Pharmaceutical Sciences, Manipal Academy of Higher Education, Manipal 576104, Karnataka, India; 2 Department of Pharmaceutical Chemistry, Manipal College of Pharmaceutical Sciences, Manipal Academy of Higher Education, Manipal 576104, Karnataka, India; 3 Manipal Centre for Biotherapeutics Research, Manipal Academy of Higher Education, Manipal 576104, Karnataka, India; 4 Department of Aging Research, Manipal School of Life Sciences, Manipal Academy of Higher Education, Manipal 576104, Karnataka, India

**Keywords:** andrographolide, liposomes, mannosylated chitosan, bioavailability enhancement

## Abstract

A key diterpene lactone of Andrographis paniculata, i.e., andrographolide (AG), exhibits a variety of physiological properties, including hepatoprotection. The limited solubility, short half-life, and poor bioavailability limits the pharmacotherapeutic potential of AG. Therefore, in this study we aimed to formulate and optimize AG-loaded nanoliposomes (AGL) using the Design of Experiment (DOE) approach and further modify the surface of the liposomes with mannosylated chitosan to enhance its oral bioavailability. Physical, morphological, and solid-state characterization was performed to confirm the formation of AGL and Mannosylated chitosan-coated AGL (MCS-AGL). Molecular docking studies were conducted to understand the ligand (MCS) protein (1EGG) type of interaction. Further, in vitro release, ex vivo drug permeation, and in vivo pharmacokinetics studies were conducted. The morphological studies confirmed that AGL was spherical and a layer of MCS coating was observed on their surface, forming the MCS-AGL. Further increase in the particle size and change in the zeta potential of MCS-AGL confirms the coating on the surface of AGL (375.3 nm, 29.80 mV). The in vitro drug release data reflected a sustained drug release profile from MCS-AGL in the phosphate buffer (pH 7.4) with 89.9 ± 2.13% drug release in 8 h. Ex vivo permeation studies showed higher permeation of AG from MCS-AGL (1.78-fold) compared to plain AG and AGL (1.37-fold), indicating improved permeability profiles of MCS-AGL. In vivo pharmacokinetic studies inferred that MCS-AGL had a 1.56-fold enhancement in AUC values compared to plain AG, confirming that MCS-AGL improved the bioavailability of AG. Additionally, the 2.25-fold enhancement in the MRT proves that MCS coating also enhances the in vivo stability and retention of AG (stealth effect). MCS as a polymer therefore has a considerable potential for improving the intestinal permeability and bioavailability of poorly soluble and permeable drugs or phytoconstituents when coated over nanocarriers.

## 1. Introduction

Hepatitis is the inflammation of the liver that can progress to cirrhosis and liver cancer if left untreated. Hepatitis is triggered by various agents such as viral infections, autoimmune diseases, and alcohol and drug toxicity. These conditions can be prevented by protecting the liver using various phytoconstituents such as silymarin, glycyrrhizin, andrographolide, curcumin, phyllanthin, berberine, embelin, resveratrol, acteoside, sauchinone, and asiatic acid [1]. For many years, andrographolide has been studied as a hepatoprotective and hepatostimulant agent, and it has also demonstrated antioxidant properties against a number of hepatotoxins [2]. Andrographolide (AG) is a diterpene lactone, obtained from *Andrographis paniculata*, and is widely used in Chinese, Southeast Asian and Indian systems of medicine for various pharmacological effects such as anti-cancer, hypoglycaemic, anti-bacterial, anti-inflammatory, antipyretic, antiviral, hepatoprotective, and detoxifying agents [3,4]. However, the therapeutic application of AG has shown restriction due to poor pharmacokinetic and physicochemical properties including poor oral absorption, low cellular permeability, short half-life, and instability in the GIT. The poor aqueous solubility and restricted bioavailability are due to extensive and rapid metabolism and efflux by P-glycoprotein [5]. AG is also known to show poor intestinal absorption due to the presence of sugar moieties that increase its hydrophilicity or bacterial degradation of its phenol moiety and complex formation with the GIT contents [3]. Nevertheless, the bioavailability of the drug in the system decides its effectiveness. Therefore, the implementation of a suitable nanocarrier to encapsulate AG can offer a comparatively affordable and indigenous therapeutic option in the treatment of liver diseases.

Liposomes are among the most suitable and well-researched drug-delivery systems known because of their tunable physicochemical and biophysical properties, good biocompatibility, controlled release of drugs, and slow drug release reservoirs that prolong the drug action via endocytosing or phagocytosing of the more vascular liver cells [6,7]. Previous studies have confirmed that when AG was loaded into the liposomes, they showed enhanced absorption, permeability and bioavailability of AG as well as improved hepatoprotective action compared to plain AG. However, liposomes can undergo chemical and physical degradation, leading to instability when given orally. The bile salts, pH, and enzymes in the GIT destabilizes the liposomal membrane. This can be prevented by coating the surface of the liposomes with natural polymers like chitosan to improve its stability [8].

Chitosan is a natural, biocompatible, non-toxic, biodegradable, and mucoadhesive polymer that can bind to the negatively charged surface of the liposome via electrostatic deposition, creating a positively charged complex with the liposome [9]. This helps the transport of the molecules through the tight junctions between epithelial cells via the interaction of negatively charged cell membranes [10]. Although chitosan coating on the liposomes facilitates the transport of drug molecules through gut epithelia, it can be further modified for better absorption, solubility, permeation, and longer circulation in the blood. This modification can be achieved through simple synthetic techniques and by modification through its amino and hydroxyl groups. One such modification was done in this study by mannose. Mannose sugar shows a significant role in metabolism along with glycosylation. The hydrophilicity of the molecule is imparted by the hydroxyl groups present on mannose and may show stealth properties when implanted onto the nanocarriers. This makes mannose a promising approach for developing mannose-grafted nanocarriers with enhanced mucopermeability [11]. Mannosylation of the chitosan can help to target the mannose receptors present in the intestinal membrane and liver which will further improve the permeation and bioavailability of AG, respectively.

**Figure 1:**
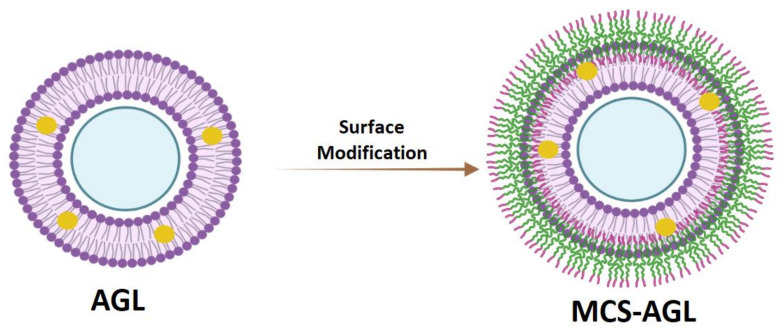
Pictorial representation of surface modification of AGL.

This study aims to prepare and optimize AG-loaded nanoliposomes (AGL) and further modify the surface with mannosylated chitosan (MCS-AGL; Figure 1) to enhance the bioavailability of AG.

## 2. Materials and Methods

### 2.1. Materials

Andrographolide (AG) was purchased from TCI, Tokyo, Japan. Chitosan, D-Mannose, Cholesterol, Sodium triacetoxyborohydride, and Soyabean Phosphatidylcholine (concentrate of soyabean lecithin consisting of > 94% of phosphatidylcholine and < 2% triglycerides) were procured from Sigma Aldrich, Bangalore, India. All other chemicals used were of analytical grade.

### 2.2. Methods

#### 2.2.1. Preparation of Andrographolide Loaded Nanoliposomes (AGL)

AGL was formulated using the thin film hydration method. Briefly, SPC, Cholesterol and AG were accurately weighed in a dried round-bottom flask and completely dispersed in the mixture of chloroform and methanol (4:1 ratio). The organic solvents were evaporated under vacuum using a rotatory evaporator at 40 °C and 200 rpm for the formation of a thin lipid film. The obtained film was kept in a vacuum oven overnight to remove any traces of the organic solvent. The film was then hydrated with phosphate buffer (pH 7.4) for 60 min at 60 °C. The formulation was then subjected to probe sonication (LABMAN PRO650, India; probe Φ3 (3 mm)) for 10 min at 40% sonication amplitude to reduce the vesicle size [12].

#### 2.2.2. Formulation Optimization Using DoE Software

The formulation was optimized using Design Expert (version 13.0.5, StatEase^®^, Minneapolis, Minneapolis, MN, USA), where the experimental design, analysis of variance (ANOVA) study, and validation of the experimental design was performed. Box Behnken design was implemented to identify the values of the independent variables leading to the best-compromised formulation having lower particle size and higher entrapment efficiency (EE). The independent variables along with the low level (−1) and high level (+1) are shown in Table 1. Based on preliminary screening studies (Supplementary Information Sections S1.1 and S2.1), the independent variables were considered to be lipid composition, sonication time, and sonication amplitude; the response variables were particle size and entrapment efficiency, respectively [13]. Herein, 15 experiments were performed as generated by the software (Table 2).

Various trial runs were performed to obtain the formulations with desired responses and the goals were integrated into an overall desirability function. The responses obtained from the trial runs were analysed using the software, which generated a study design as well as response surface plots. The substantial effect of the variable on response regression coefficients was recognized using ANOVA. The validation of the experimental design was done by checking the percent residual limit shown in the equation below, which should be within ± 10% [7]. The predicted values in the equation represent particle size and EE, respectively.

$$Percent\;Residuals=\frac{Predicted-Actual}{Predicted}\times 100$$

#### 2.2.3. Preparation of Mannosylated-Chitosan-Coated Nanoliposomes (MCS-AGL)

The prepared optimized AGL was further coated with mannosylated chitosan (0.3%) via electrostatic interaction. Mannosylated chitosan (MCS) polymer was synthesized via a reductive amination reaction using sodium triacetoxyborohydride as the reducing agent (Supplementary Information Section S1.2) [14]. Furthermore, the formation of MCS polymer was confirmed by FTIR analysis (Supplementary Information Section S2.2). The AGL liposomes were coated by MCS by first dissolving MCS in acetic acid (0.1%) followed by dropwise addition under continuous magnetic stirring for 1 h. The MCS-AGL was stored at 4 °C until further characterizations.

#### 2.2.4. Particle Size Analysis, PDI and Zeta Potential

The particle size and zeta potential of the prepared AGL and MCS-AGL formulations were determined by the DLS (dynamic light scattering) technique and electrophoretic light scattering technique using a particle size analyzer (NanoZS, Malvern Instruments, Malvern, UK). The samples were prepared by diluting them with double-distilled water and then analyzed at room temperature.

#### 2.2.5. Entrapment Efficiency

The entrapment efficiency of AGL and MCS-AGL formulation was determined by the direct method. The separation of the unentrapped drug from the AGL was done by ultracentrifugation at 22,000 rpm for 1 h at 4 °C. Later, the pellets were separated and lysed using methanol and phosphate buffer (pH 7.4) [15]. The AG concentration was measured by the High-Performance Liquid Chromatography (HPLC) Shimadzu LC-2010CHT installed with a quaternary gradient pump (low-pressure) including a UV detector, column oven, and autosampler. The separation process was performed on a Kromasil 5 μm (250 mm × 4.6 mm) C18 column. Acetonitrile and Water (pH 4 adjusted with GAA) (35:65% *v*/*v*) were used as the mobile phase at a 0.8 mL/min flow rate. The detection was performed at 223 nm. In order to analyze the AG concentration, a linear range of 0.1–20 μg/mL was used to derive the calibration curve. The LC solution 1.24 SP1 software was employed to interpret the chromatographic data. AG entrapment in the liposomes was determined using the following equation:

$$Entrapment\;Efficiency=\frac{Drug\;obtained\;in\;pellet}{Total\;drug\;added\;in\;the\;formulation}\times 100$$

#### 2.2.6. Solid State Characterization

##### Fourier Transform Infrared Spectroscopy (FTIR)

FTIR analysis was carried out for AG, SPC, Cholesterol, physical mixture, AGL, and MCS-AGL using BRUKER-ALPHA II ATR-FTIR (Bruker, Heidelberg, Germany) spectrophotometer at the wavelength range of 4000 to 400 cm^−1^. The background spectrum of the blank well was collected before each measurement. [10].

##### Differential Scanning Calorimetry (DSC)

The thermal behaviors of AG, SPC, Cholesterol, physical mixture, AGL, and MCS-AGL were analyzed using DSC (Shimadzu-TA-60 WS Kyoto, Nagoya, Japan). The analysis was performed by placing 5 mg of the sample in an aluminum pan, which was further crimped and heated from 30 to 350 °C under a nitrogen flow of 40 mL/min at 10 °C/min scanning rate. An empty aluminum pan was used as a reference [16].

##### Powder X-Ray Diffraction (PXRD)

The powder XRD patterns of the AG, SPC, Cholesterol, physical mixture, AGL, and MCS-AGL were collected using an X-ray diffractometer (Rigaku Co., Tokyo, Japan) operated at 600 watts, with a fixed voltage of 40 kV and a fixed tube current of 15 mA. A graphite monochromator was used for X-ray diffraction and detected using a standard scintillation counter. The diffraction intensities were measured over the range of 5–80° (2θ) [17].

##### Transmission Electron Microscopy

The surface morphology of AGL and MCS-AGL was evaluated by Transmission electron microscope (TEM; FEI, Tecnai G2 Spirit Bio-Twin, Eindhoven, The Netherlands). For the analysis, one drop of the sample was placed on a clean copper grid after diluting the prepared samples with distilled water and air-dried. Then, the morphology of the liposomes was checked by visualizing the grid under a high-resolution microscope [10].

#### 2.2.7. Molecular Docking Studies

The structure of MCS was downloaded from PubChem, as reported by Arif et al. [18], and optimized using LigPrep module (version 5.5, Schrödinger, New York, NY, USA) equipped with Epik (version 5.5, Schrödinger). The protein minimization was carried out with OPLS4 force field to obtain the ionized state of the molecule. The structure of the human mannose receptor protein was fetched from Protein data bank (PDB ID:1EGG) [19,20]. Protein preparation wizard was used to refine 1EGG before optimization to add the missing hydrogen and remove the water molecule [21]. Using Maestro’s GLIDE module (Grid based ligand docking with Energetics) in XP mode, the processed structures of MCS and 1EGG were molecularly docked to obtain the Glide score equation shown below [22,23].

Glide Score = Ecoul + EvdW + Ebind + Epenalty

The Prime MM-GBSA module was used to determine the binding affinity, “ΔG”. For the ligand-protein complex and individual components, several parameters including electrostatic—packing, lipophilic, van der Waals, strain, and columbic energies were estimated. The total ΔG was computed using energy-minimized parameters, as specified in the equation below [22].

ΔG = E_(MCS-1EGG (minimized)) − E_MCS (minimized) − E_1EGG (minimized)

#### 2.2.8. In Vitro Drug Release Studies

The in vitro drug release studies were conducted using the dialysis bag method. The dialysis bag was filled with samples containing 2 mg equivalent of the drug and both the ends were tied and suspended into 200 mL of pH 1.2 and pH 7.4 comprising 0.1% of Tween 80, respectively, to maintain the sink conditions. These solutions were placed in a shaking incubator at 37 °C and 100 rpm. We withdrew 2 ml of the sample after appropriate intervals of 0.25, 0.5, 0.75, 1, 2, 4, 6, 8, and 12 h and replenished with 2 mL of fresh buffer solutions. Similarly for pH 1.2, the samples were withdrawn at 0.25, 0.5, 0.75, 1, and 2 h. The drug concentration in the solution was estimated using the HPLC method described in Section 2.2.4 [16].

#### 2.2.9. In Vitro Cell Viability Assay

This assay is a sensitive, accurate, and trustworthy colorimetric assay that evaluates the viability, proliferation, and activation of cells. The test relies on mitochondrial dehydrogenase enzymes’ ability to convert the yellow, water-soluble substrate 3-(4,5-dimethylthiazol-2-y1)-2,5-diphenyl tetrazolium bromide (MTT) into a dark blue, water-insoluble formazan product [1]. The cytotoxicity of the prepared nanoliposomes was evaluated on HepG2 cells using MTT assay. HepG2 cells were seeded in 96-well plates at a density of 105 cells per 100 μL and allowed to adhere overnight in a humidified atmosphere with 5% CO_2_. In triplicate, cells were treated with plain AG and nanoformulations (Placebo, AGL and MCS-AGL) of different doses (1 ng/mL, 10 ng/mL, 100 ng/mL, 1 μg/mL and 10 μg/mL) along with Mitomycin (10 μg/mL) as a positive control. After incubation, the supernatant was carefully discarded and MTT (100 μL) was added to wells 24 h after treatment and left for another four hours. After dissolving the formazan crystals in isopropanol, the absorbance at 545 nm was measured using a microplate reader. The percentage of cell survival was evaluated in comparison to the untreated control.

(1) $$Cell\;viability\;\left(\%\right)=\frac{Sample\;optical\;density}{Control\;optical\;density}\times 100$$

#### 2.2.10. Ex Vivo Permeation Study

The ex vivo permeation study was carried out to determine the drug absorption via the ileum portion of the small intestine using a non-everted rat ileum sac model [24]. The experiments were carried out in Wistar rats (weighing 200 ± 50 g). Before commencing the study, approval was sought from the KMC Manipal, Institutional Animal Ethics Committee (IAEC), MAHE (IAEC/KMC/28/2022). The rats were euthanized, and the intestine was isolated and cleaned with saline solution. The ileum was placed in a petri dish bubbled with oxygen and then the mucosal side was filled with 0.5 mL of drug solution, and both ends of the sac were tightly ligated. Further, the sac was immersed in 20 mL Krebs solution in a beaker. The sampling of 1 mL was obtained from the serosal medium at predefined time intervals for 180 min to determine the concentration of the drug permeated from the mucosal medium to the serosal medium. One ml of fresh Krebs solution was replenished at each interval. Later, the collected samples were centrifuged and the concentration of the drug permeated was determined by using the HPLC method. A similar study was done for AGL and MCS-AGL [17]. The apparent permeability coefficient (P_app_) was calculated conferring to the given equation and expressed in cm.min^−1^.

(2) sPapp=dQdt∗1ACo

where dQ/*dt* is the rate of drug appearance on the basolateral side, *C*_0_ is the initial concentration over the apical side, and *A* is the surface area of intestinal tissue (cm^2^).

#### 2.2.11. In Vivo Pharmacokinetics Study

The pharmacokinetics of AG were studied in Wistar rats (weighing 250 ± 50 g). Before commencing the study, approval was sought from the Institutional Animal Ethics Committee (IAEC), Manipal Academy of Higher Education, Manipal (IAEC/KMC/28/2022). The animals were handled according to the institutional and national guidelines for the use and care of animals. The rats were divided into 3 groups (*n* = 4) (AG dispersion, AGL, and MCS-AGL). The rats were dosed at 50 mg/kg of AG by oral gavage with respective formulations. Blood samples (0.3 mL) were withdrawn from the retro-orbital venous plexus into 2 mL tubes containing EDTA solution at 0.5, 1, 2, 4, 8, 12, and 24 h after oral administration. The blood samples were immediately centrifuged at 10,000× *g* rpm for 10 min in a cooling centrifuge to separate the plasma [15].

The AG concentration in rat plasma was estimated using HPLC-UV. The calibration curve was developed with a linear range of 25 to 5000 ng/mL. The liquid–liquid extraction method was utilized for extracting AG from the rat plasma. Carbamazepine was used as the internal standard at a concentration of 10 μg/mL. An amount of 80 μL of the sample was injected in HPLC for analysis. The samples were eluted using the method described in Section 2.2.4. The pharmacokinetics parameters were calculated using PK solution software (PK Solutions 2.0^TM^). The pharmacokinetic parameters included maximum plasma concentration (C_max_), area under the plasma concentration-time curve (AUC), elimination half-life (K_el_), absorption half-life (t_1/2_), and mean residence time (MRT).

## 3. Results

### 3.1. Formulation and Optimization of AGL

The thin film hydration technique is the most commonly used approach for the preparation of liposomes. However, various variables affect the process using a rotary evaporator which needs to be optimized. Various trials were performed to optimize the temperature of the water bath and the speed of rotation of the RBF to form a thin and continuous film. A preliminary study was conducted to screen the variable affecting the formulation. This preliminary study (Supplementary Information Section S2.1) confirmed that the variables influencing the formulation were lipid concentration, sonication amplitude, and sonication time, which were further considered for the optimization process [25].

**Figure 2:**
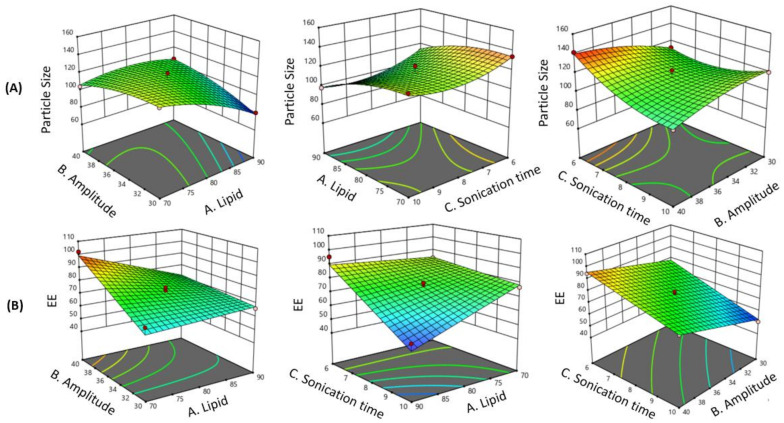
The 3D surface plots indicating the effect of variables on particle size and EE. (**A**): Particle size; (**B**): EE.

The AGL formulation was optimized using the Box Behnken design model pertaining to particle size and % EE. The results obtained after 15 experiments are stated in Table 2. The results depicted the model to be significant with respect to particle size and % EE because the *p*-value was found to be less than 0.05. To determine the ideal experimental parameter, all observed responses were evaluated in comparison. ANOVA method was used to determine the finest mathematical model and optimized parameters by analyzing the responses. The *p*-values of individual and combined variables proved the effect of a respective variable on selected responses. The *p*-values also highlighted the significance of the applied model and other parameters evaluated by ANOVA. The model was found to be significant, while the lack of fit was not significant for each observed response. The 3D response surface plots of all the responses indicating the influence of various variables are represented in Figure 2. The final regression equation of the model for particle size and entrapment efficiency produced by the software are given below:

(A) Particle size (nm) = −48.00208 + 2.49021 * Lipid + 5.18208 * Amplitude + 5.51333 * Sonication time + 0.223000 * Lipid*Amplitude − 0.079875 * Lipid * Sonication time − 1.12725 * Amplitude*Sonication time − 0.067408 * Lipid − 0.192833 * Amplitude + 2.31667 * Sonication time
(B) EE (%) = −566.84200 + 8.21825 * Lipid + 13.31600 * Amplitude + 22.95625 * Sonication time − 0.15560 * Lipid * Amplitude − 0.419750 * Lipid*Sonication time + 0.141000 * Amplitude * Sonication time

The response surface plots and coefficient of the quadratic equation firmly defined that the selected variables considerably influence the dependent variables.

### 3.2. Effect of Independent Variables on Particle Size

The results indicated that the particle size values of all the prepared formulations range from 80 nm to 141 nm, suggesting that the independent variables had a substantial influence on the particle size of AGL. From the ANOVA results (Table 3), it is evident that the independent variables, i.e., the lipid concentration (*p*-value: 0.0010), and sonication time (*p*-value: 0.0107) had a significant impact on particle size. The r^2^ value was found to be 0.9656 and the Adjusted r^2^ of 0.9036 indicated that the model is appropriate to navigate the design space. The regression equation distinctly indicates that the lipid concentration, sonication time, and amplitude directly affected the particle size of the liposomes. The 3D surface plots indicating the correlation between the input variables and particle size are shown in Figure 2. The particle size of the liposomes was found to be significantly influenced by lipid concentration. These figures suggest that as the concentration of lipid content increases, there is a decrease in the particle size. Similarly, the particle size of the liposomes tends to increase with increase in sonication amplitude. Higher sonication amplitude causes stronger agitation on the particles further breaking the lipid layer. In our work at 30% amplitude, multilamellar liposomes were reduced to unilamellar liposomes; however, with a further increase in amplitude, the lipid layer of the unilamellar liposome were degraded, leading to immature particles and thus causing the increase in particle size and PDI due to agglomeration. The results suggested that sonication time showed a curvilinear decrease pattern on the particle size, i.e., initially, the particle size decreased with increasing time but after a point, the particle size again increased. This reduction in the particle size with an increase in the lipid concentration can be a result of the higher solubility of phospholipids at the interface of the two phases [26].

#### 3.2.1. Effect of Independent Variables on Entrapment Efficiency

The EE of liposomes varied from 50% to 96%, indicating that the independent variables had an impact on the EE, and the ANOVA results suggested a significant model (Table 3). All the independent variables, i.e., lipid concentration, amplitude, and sonication time, showed significant influence on the % EE. The r^2^ value was found to be 0.9292 and the adjusted r^2^ of 0.9036 indicates that the model is appropriate to navigate the design space. The regression equation clearly indicated that the lipid concentration, amplitude, and sonication time directly affected the EE of the liposomes. The 3D surface plots indicating the correlation between input variables and % EE are shown in Figure 2. These figures suggest that with the increase in the amount of lipid, the EE continues to decrease. Similarly, with an increase in the sonication time, the EE of the drug decreases, which shows a significant effect of sonication time on the EE of liposomes. The drug EE of the liposomes goes on increasing with an increasing amplitude, which also indicates that amplitude has a significant effect on EE. Higher sonication time causes disruption of the lipid bilayer of the liposomes, resulting in drug leakage, thus reducing the EE [27].

#### 3.2.2. Validation of BBD Model

The numerical optimization was performed by establishing the goals for the responses to produce the perfect conditions. To achieve this, the particle size was selected to be minimum and the % EE was selected to be maximum. After establishing the result, it was validated pertaining to particle size and % EE to ascertain the robustness of the implemented design, as shown in Table 4. The particle size achieved for the validation was within the expected range of around 85 nm, with an EE of about 90%, as shown in Table 4. These results were consistent with the predicted responses, demonstrating the robustness of the design for the preparation of liposomes.

### 3.3. Particle Size Analysis, PDI, Zeta Potential and Entrapment Efficiency

The particle size, PDI, and zeta potential of the optimized AGL formulation were found to be 86.60 nm, 0.215, and −67.7 mV, respectively. The coating of the liposomes with MCS showed an increment in the size of the liposomes. This increase in size is due to electrostatic interaction, which results in the formation of a bridge between the MCS and the surface of the liposome. After coating, the particle size of the liposomes increased to 375.3 nm, suggesting the successful MCS coating on the plain liposomes. Several studies have found that the formation of polymer coating on the liposomes is confirmed by the inversion of the zeta potential from negative to positive values between the uncoated and coated systems [28]. Similarly, we found that the zeta potential changed from −59.6 mV to 29.8 mV. The MCS carried a high positive charge, due to which the adsorption of MCS on the negatively charged surface of the liposomes increased the density of the positive charge and made the coated liposomes positive. Even though the MCS coating broadened the mean particle size of the liposomes, the PDI values remained below 0.3, indicating an acceptable degree of polydispersity [28]. The entrapment efficiency of the liposomes is calculated to determine the amount of drug in the liposomes. The EE was determined using HPLC. The standard calibration curve over the AG concentration range of 100–20,000 ng/mL was found to be linear with the regression coefficient (r^2^) value of ≥ 0.999. Here, the EE of AGL was found to be 90.06%. High drug entrapment could be due to the high affinity of the drug with SPC, which entraps the drug into the lipophilic layer of liposomes [10], while a slight decrease in the EE of the MCS-AGL (81.41%) could be the result of the drug leaking from the liposomes during the 1 h stirring process that occurs during coating.

### 3.4. Solid State Characterization

#### 3.4.1. FTIR Studies

**Figure 3:**
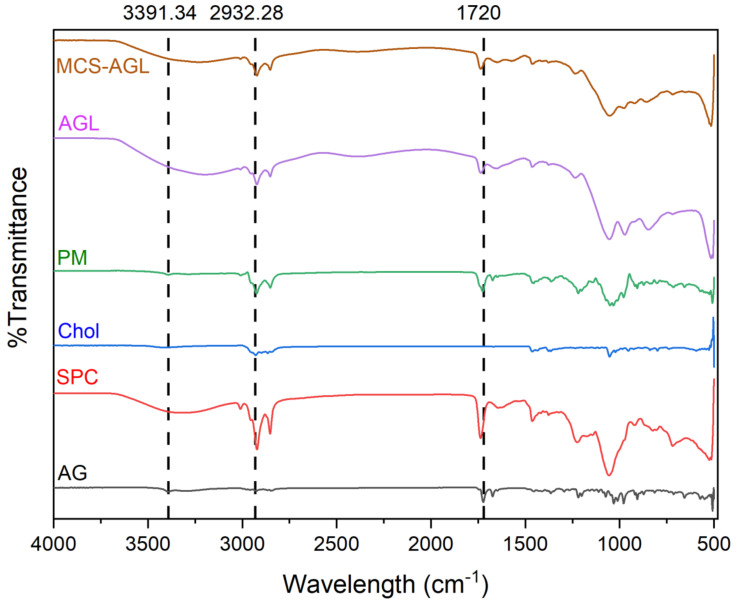
FTIR spectra of pure AG, SPC, Cholesterol, physical mixture, AGL, and MCS-AGL.

The FTIR spectra of the AG, physical mixture, AGL, and MCS-AGL were carried out to investigate any potential interactions between the excipients and the drug. The spectrographs of AG, SPC, cholesterol, physical mixture, AGL, and MCS-AGL formulation are illustrated in Figure 3. The spectrum of AG showed peculiar peaks of the functional group -OH at 3391.34 cm^−1^, 3302.05 cm^−1^, -C=O at 1720 cm^−1^, and C-H at 2800–3000 cm^−1^ [7]. SPC and cholesterol showed peaks between 3350–3450 cm^−1^, which indicates the stretching vibration of free and bonded hydroxyl (OH) and amine (NH2) groups. Cholesterol also showed a peak at 2932.28 cm^−1^, indicating CH2 and CH3 groups. The characteristic peaks between 3350–3450, 2923, 1729, and 2800–3000 cm^−1^ in the physical mixture confirm the presence of SPC, cholesterol, and AG in the mixture. The spectrum of the physical mixture differs substantially compared to the spectrum of AG and SPC, indicating the reaction of the -OH group of AG with the choline group of SPC, which confirmed the complex formation [29]. All the results obtained comply with the results obtained from previous studies. Overall, the results suggested compatibility between AG and excipients. Due to the formation of the lipid and cholesterol vesicle, the peak size, shape, and intensity of AG were found to be reduced. However, some peaks of AG were found to have disappeared compared to the plain AG spectrum, showing the entrapment of AG into the lipids [29]. The IR spectra of MCS-AGL showed further reduction in the size and shape of the peaks, confirming the surface coating of the liposomes. The MCS-AGL FTIR spectrum resembles the spectrum of MCS polymer (Supplementary Information Section S2.2 (Figure S1)), confirming the coating of MCS polymer on the surface of the liposomes.

#### 3.4.2. DSC Studies

**Figure 4:**
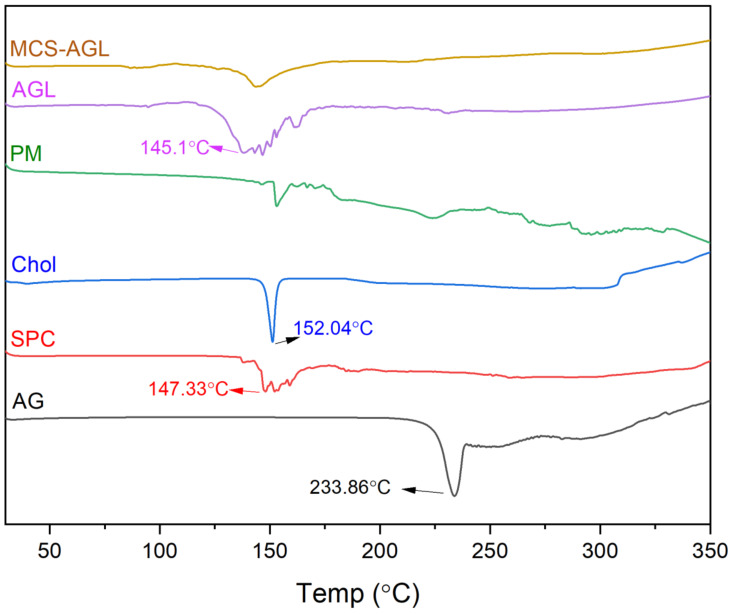
DSC thermogram of pure AG, SPC, Cholesterol, physical mixture, AGL, and MCS-AGL.

DSC analysis was carried out to check the thermostability and the physical nature of the compound. The thermograms of AG, SPC, Cholesterol, physical mixture, AGL, and MCS-AGL are depicted in Figure 4. The thermogram of AG illustrated a peak at 233.86 °C, which indicated its melting point. SPC showed a mild peak at 147.33 °C, which is possibly owing to the crystal–liquid phase transition. The cholesterol thermogram showed a peak at 152.04 °C. The results obtained comply with the results acquired from previous studies [29]. The thermogram of the physical mixture of SPC, cholesterol, and AG showed two different peaks at 151.07 °C, 173.22 °C, and 225.80 °C, which are predicted to be of SPC, cholesterol, and AG, respectively. The perturbing effect on the peak indicates the interaction of cholesterol with SPC. However, these peaks indicated that the excipients did not show any interaction with the drug, hence the drug and the excipients are compatible. The sharp peaks of AG and cholesterol show the crystalline nature of the compounds while the broad peaks of SPC confirm its amorphous nature. The thermogram of AGL illustrated a broad peak at 145.10 °C, which is due to the complex formation between SPC and cholesterol. The peak of AG was not observed in AGL, which indicates that the drug has been successfully entrapped into the liposomes. Similarly, the thermogram of MCS-AGL shows similarities with the thermogram of AGL, but the change in the shape of the peak can be due to the presence of mannose, and the peak at 87 °C is of chitosan, as indicated in Supplementary Information Section S2.2 (Figure S2). The change in the shape of the peak from broad to sharp can be due to the presence of crystalline mannose, indicating the surface coating of mannose. The chitosan peak has also shown a reduction in size, possibly due to the interaction of chitosan with phospholipids.

#### 3.4.3. XRD Studies

**Figure 5:**
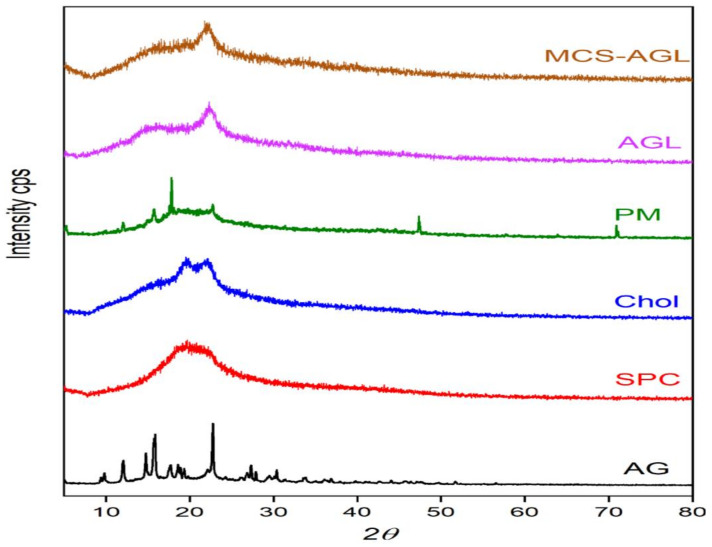
XRD diffraction patterns of pure AG, SPC, Cholesterol, physical mixture, AGL, and MCS-AGL.

X-ray diffraction analysis allows us to determine the crystalline properties of the raw materials and the liposomes. The XRD diffraction patterns obtained are shown in Figure 5. The diffraction pattern of AG was highly crystalline in nature, as indicated by numerous peaks. In contrast, SPC showed a broad peak with a diffraction pattern at 20°, indicating a comparatively less crystalline nature. From the diffraction pattern of the physical mixture, the presence of a few sharp peaks confirms the existence of AG in the mixture, and the disappearance of a few characteristic peaks confirms the formation of an amorphous polymer, which may be with intermolecular interaction of AG with SPC. Similarly, the diffraction patterns obtained for AGL consisted of overlaps of spectra obtained from the pure components. The data suggested that changes in the physical state of AG confirm the loss of crystallinity following encapsulation in liposomes. Okafor et al. also portrayed similar kinds of results for efavirenz liposomes [10]. Pure d-mannose showed a crystalline nature due to the presence of numerous sharp peaks; pure chitosan exhibited two broad peaks at 2θ = 14° and 21°, indicating its amorphous nature [11]. The change in the shape of the peak was observed from the MCS polymer diffraction pattern, which may indicate the formation of a complex between mannose and chitosan. The presence of a few peaks in the polymer indicated the presence of mannose. The diffraction peak of MCS-AGL shows similarity with the diffraction peak of AGL and MCS polymer. The broad peak confirms the amorphous nature of the formulation.

#### 3.4.4. Transmission Electron Microscopy

**Figure 6:**
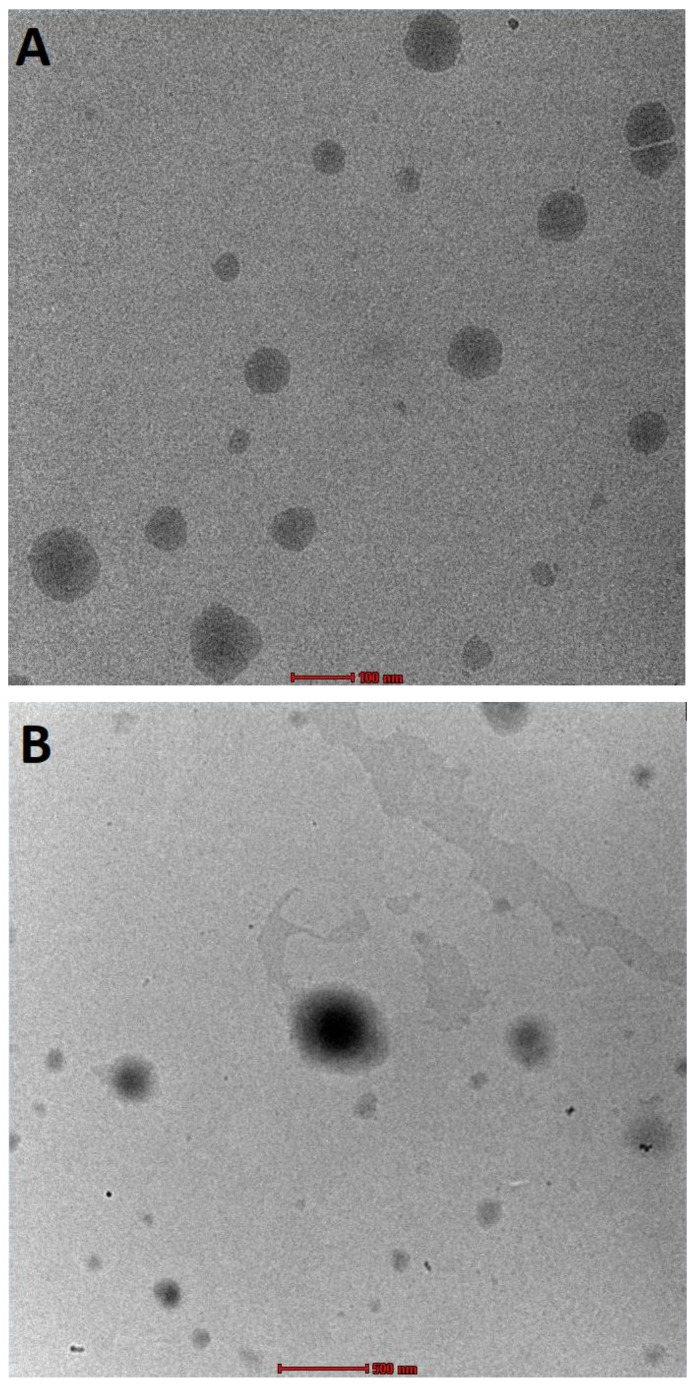
TEM images of andrographolide liposomes (AGL) and Mannosylated chitosan-coated andrographolide liposomes (MCS-AGL). (**A**): AGL at 100 nm scale; (**B**): MCS-AGL at 500 nm.

The TEM images (Figure 6) showed nanosized spherical vesicles of both AGL and MCS-AGL. No considerable difference except for particle size was observed between the uncoated AGL and coated MCS-AGL. Figure 6A confirmed the spherical morphology of the liposomes. A lipophilic layer could be clearly observed in the image. DLS measures particle diffusivity and corresponds to the hydrodynamic diameter, which is the diameter of the particle and the surrounding observable layer. TEM analysis measured the diameter of a dry or dishydrated particle [30]. However, the size of the spherical liposomes was found to be in correspondence to the particle size found by the light scattering method by NanoZS (Malvern Instruments, Malvern, UK). The existence of MCS coating surrounding the liposomes was clearly observed in Figure 6B. It was also confirmed that the coating of the liposomes with MCS did not alter the shape of AGL.

### 3.5. Molecular Docking

**Figure 7:**
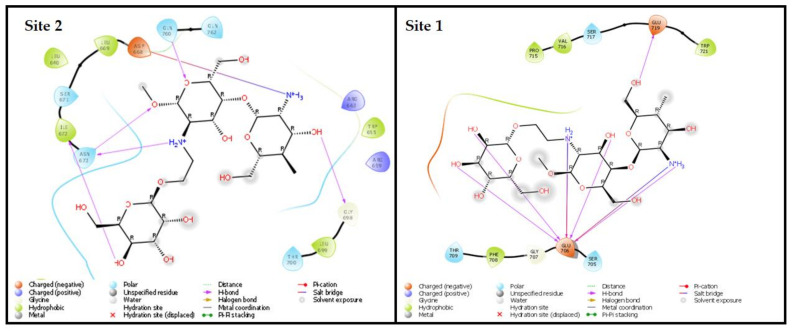
In silico molecular docking 2D images of ligand protein contact; sites 1 and 2.

The approach of using in silico tools to forecast targeting efficiency before any experimental investigation is critical to the pharma industry [1]. The goal of the molecular docking study was to comprehend the potential interactions with 1EGG and MCS. The best two docking positions (site 1 and site 2) demonstrated an excellent fit for the MCS within IEGG (Figure 7). According to the findings, hydrogen bonds are primarily responsible for the host–guest (1EGG-MCS) molecular interaction. When docked with the 1EGG protein at site 1, the MCS complex exhibits numerous interactions (hydrogen bond and salt bridge) with the GLU 706 amino acid and one hydrogen bond interaction with the GLU 719 amino acid, both with a docking score of −5.866 and an MMGBSA dG bind score of −31.54. However, various amino acids interacted with the MCS, including ASP668 (salt bridge), ILE672, ASN673, GLY698, and GLN760 (hydrogen bond) when MCS docks at site 2, giving the complex a docking score of −5.205 and an MMGBSA dG bind score of −37.78. Moving further to our research, the molecular docking study predicted the targeting capability of MCS-AGL. The molecular docking analysis gave us a good docking score with 1EGG. Sites 1 and 2 showed a good docking score; however, site 2 showed interactions with different amino acids, which demonstrates a substantial attachment of MCS to site 2 compared to site 1 of the 1EGG. The findings indicated that the MCS complex, by interacting primarily at site 2, might be a possible option for targeting the mannose receptor.

### 3.6. In Vitro Drug Release Studies

**Figure 8:**
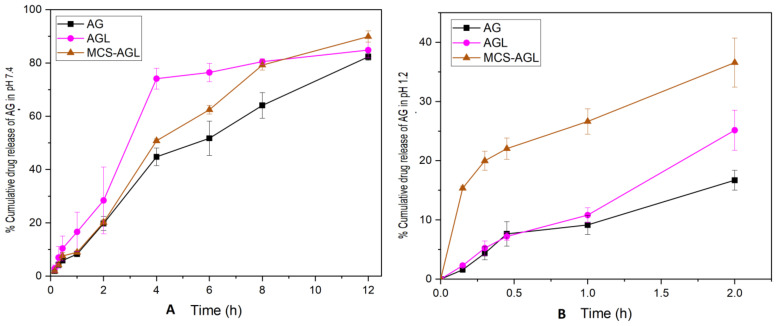
In vitro drug release study of AG, AGL, and MCS-AGL. (**A**) represents the in vitro drug release in pH 7.4. (**B**) represents the in vitro drug release at pH 1.2. The results are expressed as mean ± SD, *n* = 3.

The in vitro drug release studies for plain AG, AGL, and MCS-AGL were carried out using a dialysis membrane in pH 1.2 HCl solution and phosphate buffer of pH 7.4 to mimic the gastric environment and intestinal environment, respectively. The drug release was assessed from the outer bulk solution over a period of time. In vitro AG release profiles from AG dispersion, AGL, and MCS-AGL in phosphate buffer pH 7.4 for 12 h and HCl buffer for 2 h are shown in Figure 8A and Figure 8B, respectively. The AG suspension, AGL, and MCS-AGL showed 16.7 ± 1.66%, 25.14 ± 3.40%, and 36.56 ± 4.17% drug releases, respectively, in 2 h in the gastric environment. The initial higher drug release of AGL was due to the presence of AG at the surface of the vesicles and it was instantly available for the release media. In contrast, MCS-AGL showed higher drug release in pH 1.2 because the amino groups in the chitosan become protonated in the acidic medium, and the polymer dissolves. However, the drug release in phosphate buffer pH 7.4 from AGL and MCS-AGL showed 84.83 ± 2.93% and 89.9 ± 2.13% drug release, respectively. MCS-AGL showed a sustained drug release pattern [31]. The variation in release patterns between AGL and MCS-AGL can be associated with the variations in structural integrity deterioration of the AGL and MCS-AGL [10].

### 3.7. In Vitro Cell Viability

**Figure 9:**
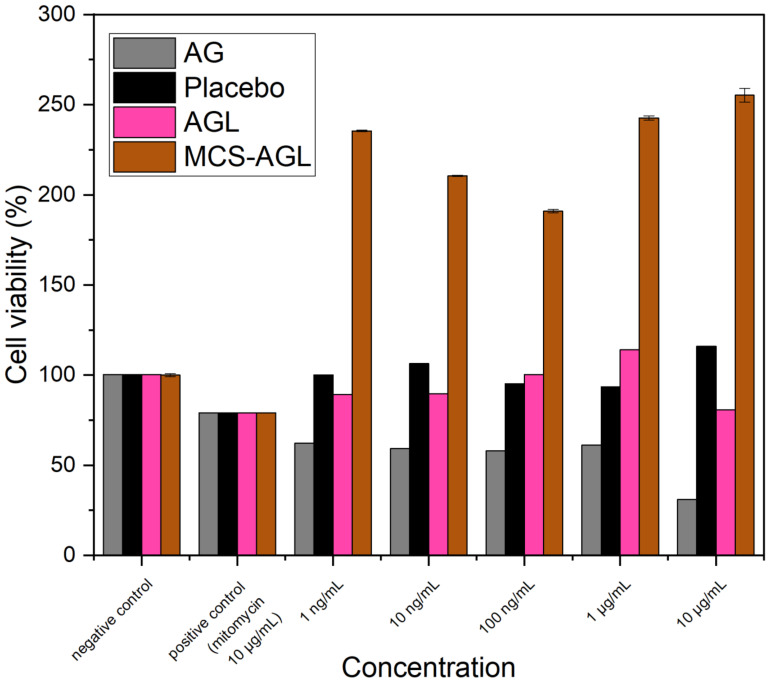
In vitro cell viability of plain AG, positive control mytomycin (10 μg/mL), placebo liposomes, AGL, and MCS-AGL on HepG2 cells according to MTT assay after 24 h. The results are expressed as mean ± SD, *n* = 3.

Figure 9 illustrates the results of the MTT assay, which was used to determine the viability of the HepG2 cells after 24 h of incubation with plain AG, positive control mytomycin (10 μg/mL), placebo liposomes, AGL, and MCS-AGL. Based on the MTT data, the results of the placebo liposomes showed 100% cell viability, which denotes that the nanocarrier was not toxic. The cell viability decreased to about 60% at all concentrations examined when the cells were treated with plain AG, indicating a small extent of cytotoxicity with pure AG [2,3,4]. Improved cell viability was observed with AGL in comparison with plain AG, demonstrating that the cytotoxicity of the drug was reduced when loaded into a nanocarrier. An interesting observation with MCS-AGL was that the cell viability increased twofold at all concentrations examined, suggesting cell proliferation. To further understand the proliferation mechanism and to aid in liver regeneration or the treatment of any liver illnesses, this polymer characteristics can be investigated further [5].

### 3.8. Ex Vivo Permeation Study

**Figure 10:**
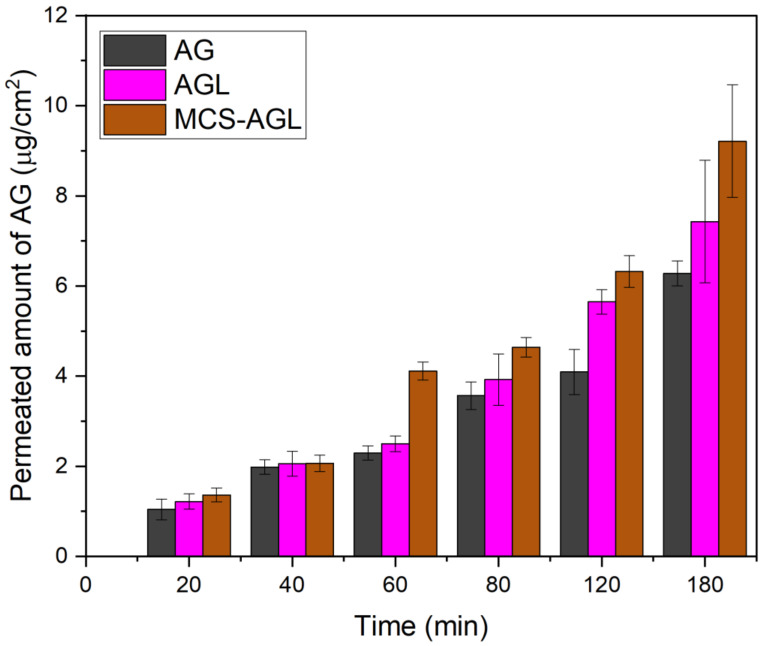
Ex vivo permeation studies of AG, AGL, and MCS-AGL. The results are expressed as mean ± SD, *n* = 3.

The permeation studies were performed using a noneverted rat ileum sac model. The apparent permeability of plain AG dispersion, AGL, and MCS-AGL was found to be 1.42 × 10^−2^ cm/min, 2.36 × 10^−2^ cm/min, and 3.34 × 10^−2^ cm/min, respectively. This indicated that AGL and MCS-AGL showed a 1.66-fold and 2.35-fold enhancement in the permeation compared to pure AG, respectively. Figure 10 gives detailed information about the permeation study of AG, AGL, and MCS-AGL at different time points. The permeated amount of AG was plotted against time. It was found that AGL showed higher permeation compared to AG, but lesser than MCS-AGL. As liposomes are known to show enhanced permeation through the intestine, higher permeation of AGL was probably due to the lipidic nature of the liposomes and the interaction of phospholipid head groups with mucous glycoproteins of the intestine. MCS-AGL displayed higher permeation of AG in comparison to AG dispersion and AGL. The mannose receptor present in the intestine plays an important role in the permeation of MCS-AGL. The permeation enhancement may be due to the interaction of MCS with the mannose receptors present in the intestine. The intestinal membrane is covered with negatively charged sialic acid where the positively charged chitosan can easily bind due to electrostatic attraction. Therefore, it shows higher permeation through the mucosal surface [10].

### 3.9. In Vivo Pharmacokinetics Study

**Figure 11:**
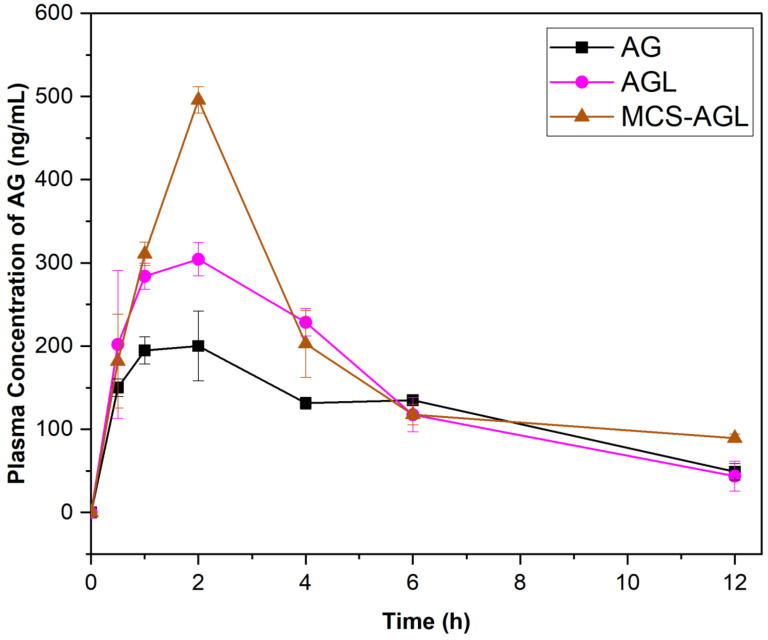
Pharmacokinetics profile of AG, AGL, and MCS-AGL.

The pharmacokinetic study was examined on Wistar rats weighing around 250 ± 25 g. The rats were administered AG dispersion, AGL, and MCS-AGL at 50 mg/kg dose by oral gavage. The standard calibration curve for AG in rat plasma over the AG concentration range of 100–20,000 ng/mL was obtained to be linear with the regression coefficient (r^2^) value of around 0.999. The plasma concentration–time profiles of AG in all three groups were established, and the acquired data is illustrated in Figure 11. The pharmacokinetics parameters are illustrated in Table 5. The oral administration of AGL and MCS-AGL led to a substantial increase in C_max_, T_max_, AUC_0–24_, and AUC_0–∞_ relative to the corresponding pharmacokinetic parameters of AG dispersion. The AUC and C_max_ of MCS-AGL were higher than that of AG dispersion and AGL, while the elimination of MCS-AGL from the plasma is lower than AGL and AG dispersion. This showed that the release rate of AG was relatively slow from MCS-AGL, thus it will exhibit a sustained effect. The sustained-release of AG has a good effect on extending the duration of pharmacological activity in vivo and, as a result, lowering the frequency of dose in clinical therapy. As shown in Figure 9, the AG plasma concentrations were significantly higher for rats treated with MCS-AGL than the rats treated with AGL and AG dispersion. AGL-treated animals showed significantly higher plasma concentrations than AG-dispersion-treated animals. The Cmax value of MCS-AGL, AGL, and AG are 495.90 ± 15.78 ng/mL, 310.03 ± 12.64 ng/mL, and 207.14 ± 35.59 ng/mL, respectively. The AUC of AG dispersion, AGL, and MCS-AGL were 1410.3 ± 84.40 ng/mL*h, 1829.97 ± 141.66 ng/mL*h, and 2213.46 ± 50.05 ng/mL*h, respectively. The results demonstrated that the AUC of MCS-AGL was 1.2-fold of AGL and 1.3-fold of AG dispersion. This proved that the MCS coating significantly enhanced the bioavailability of AG in plasma. The hydroxyl groups on mannose contribute hydrophilicity to the molecule, which may have stealth characteristics when grafted onto nanocarriers to produce mannose-grafted nanocarriers with improved mucopermeability. The results suggest the presence of mannose receptors in the intestinal membrane and liver, which attracted the positively charged mannosylated chitosan and increased its absorption. Consequently, these results suggest that the MCS-AGL enhanced the oral bioavailability of AG and provided a prolonged AG release after oral administration in rats.

## 4. Conclusions

The current research was focused on enhancing the oral bioavailability of AG by developing MCS-coated liposomes. AGL were successfully developed using a thin film hydration method and optimized using Box Behnken design (BBD) to achieve desired target attributes. MCS coating on the liposomal surface was successfully formulated and analyzed using the aforementioned characterization techniques. The optimized liposomes had high drug entrapment and particle homogeneity. The in vitro drug release studies confirmed the sustained release pattern of MCS-AGL in the intestinal pH. The in vivo pharmacokinetics study and ex vivo permeation study proved the enhanced permeation of AG through MCS-AGL, thereby enhancing oral bioavailability The results indicated that the mannosylated chitosan coating on nanocarriers can prove to be a potential alternative to improve the permeation of lipophilic drugs and facilitate the absorption of liposomes through the intestine, thus improving its bioavailability. Additional research involving pharmacodynamic tests and in vitro hepatoprotective tests in a suitable animal model will be necessary to conduct future studies to determine the therapeutic efficacy of AG in the optimized formulations. Considering the complexity of the method of preparation, further investigations are required to ease the scale up of the coated nanoformulations.

## Figures and Tables

**Table 1:** Experimental design and variables.

Factors	Name/Variables	Units	Levels	
			Low Level (−1)	High Level (+1)
A	Lipid	mg	70	90
B	Amplitude	%	30	40
C	Sonication time	min	6	10

**Table 2:** Observed responses in BBD during optimization of independent variables involved in the preparation of AG-loaded liposomes.

Run	Factor 1 A: Lipid (mg)	Factor 2 B: Amplitude	Factor 3 C: Sonication Time (min)	Response 1 Particle Size (nm)	Response 2 EE (%)
1	70	35	10	126	76.8
2	90	35	6	112.2	95.16
3	90	40	8	106.33	68.48
4	70	40	8	104.3	96.1
5	80	35	8	112.23	78.31
6	80	30	10	120.03	50.6
7	90	30	8	80.23	66.1
8	80	40	10	103.8	75.4
9	80	30	6	112.5	75.2
10	80	35	8	122.5	75.82
11	80	40	6	141.36	94.36
12	90	35	10	97.91	54.64
13	70	35	6	133.9	83.74
14	70	30	8	122.8	68.6
15	80	35	8	110.2	67.01

**Table 3:** ANOVA table for Quadratic model of two studied responses (particle size and Entrapment efficiency).

Content	Particle Size		Entrapment Efficiency	
Source	*p*-value	Status	*p*-value	Status
Model	0.0038	Significant	0.0003	Significant
A-Lipid	0.0010		0.0108	
B-Amplitude	0.1853		0.0005	
C-Sonication time	0.0107		0.0002	
AB	0.0049		0.0146	
AC	0.5233		0.0101	
BC	0.0047		0.5892	
Lack of Fit	0.9115	Not significant	0.7261	Not significant

**Table 4:** Actual and predicted values of optimized formulation.

	Independent Variables			Responses	
	Lipid (mg)	Amplitude (%)	Time (min)	Particle Size (nm)	Entrapment Efficiency (%)
The composition suggested by the software	90	30	6	84.65	88.61
Practically performed composition	90	30	6	86.60	90.06
Residual error (%)	-	-	-	−2.29	−1.63

**Table 5:** Pharmacokinetic parameters of AG, AGL and MCS-AGL.

Parameters	AG	AGL	MCS-AGL
t_1/2_ (h)	4.06 ± 1.10	4.23 ± 1.13	16.17 ± 4.36
Tmax (h)	1.33 ± 0.57	1.5 ± 0.86	2.00 ± 0.00
Cmax (ng/mL)	207.14 ± 35.59	310.03 ± 12.64	495.90 ± 15.78
AUC_0–24_ (ng/mL*h)	1410.3 ± 84.40	1829.97 ± 141.66	2213.46 ± 50.05
AUC_0–I_ (ng/mL*h)	1869.20 ± 170.47	2116.78 ± 317.46	4298.36 ± 580.31
MRT (h)	8.6 ± 0.66	5.93 ± 1.53	19.43 ± 4.84
Ke (h^−1^)	0.13 ± 0.022	0.21 ± 0.022	0.09 ± 0.09

## Data Availability

The data may be available on request.

## Supplementary Materials

The following supporting information can be downloaded at: https://www.mdpi.com/article/10.3390/membranes13020193/s1, Figure S1: FTIR spectra of D-mannose, Chitosan, and MCS polymer title; Figure S2: DSC thermogram of D-mannose, Chitosan, and MCS polymer; Table S1: Optimization of rotary evaporator parameters; Table S2: Optimization of probe sonicator parameters.
