# Supplementary material for: Mannosylated-Chitosan-Coated Andrographolide Nanoliposomes for the Treatment of Hepatitis: In Vitro and In Vivo Evaluations
Source: Membranes (Basel). 2023 Feb 3;13(2):193. doi: 10.3390/membranes13020193 (PMC9965523; doi:10.3390/membranes13020193)
Supplement: Supplementary file 1 [file membranes-13-00193-s001.zip › membranes-2134487-supplementary.pdf]

Supplementary data

S1. Methods

S1.1 Preliminary screening studies using one factor at a time method

a. ***Selection of lipids***

For the selection of lipids, various trials were performed using different lipids to prepare the thin film. The lipid which formed a proper continuous thin film was selected. SPC, Phospholipon 80 M and Lipoid S100 were tried.

b. Selection of rotary evaporator parameters

The temperature of the water bath and speed of rotation of the flask were the parameters selected for screening its effect on thin film preparation. Different temperatures like 45 °C and 40 °C were tried and 100, 150 and 200 RPM were tried.

c. Selection of probe sonicator parameters

Sonication time, Sonication amplitude, and Pulse “on-off” time were the parameters selected for screening its effect on the particle size of liposomes. the trials were performed at 5 mins and 10 mins with 20 and 40% amplitude and 4-2 and 10-2 pulse on and off time.

S1.2 Preparation of mannosylated chitosan (MCS) polymer

The MCS polymer was synthesized via a reductive amination reaction using sodium triacetoxyborohydride [14]. Briefly, 500 mg chitosan was dissolved in 30 ml of acetic acid (0.1 mol/L) to form a solution by stirring overnight at 1000 rpm on a magnetic stirrer at 60°C. Later the pH of the solution was adjusted to 4.5 using 0.1M NaOH. Another solution was prepared using 0.675 g D-mannose and 0.75 g sodium triacetoxyborohydride in 10 ml of distilled water. Later these two solutions were mixed at room temperature for 48 hours at 400 rpm on a magnetic stirrer to acquire a thick solution. Lastly, using double-distilled water the thick solution was dialyzed using a dialysis membrane. The obtained solution was lyophilized to obtain the complex of MCS.

S2. Results and Discussion

S2.1 Preliminary screening studies

a. Selection of lipids

Three batches of liposomes were prepared with different lipids including SPC, phospholipon 80H, and Lipoid S100 keeping all other variables constant where SPC formed a proper and continuous film as compared to other lipids. Hence SPC was selected for the formulation.

b. Selection of rotary evaporator parameters

Based on the observations mentioned in Table S1, 40 °C temperature and 200 RPM formed the proper film. Hence these parameters were considered for further optimization.

Table S1: Optimization of rotary evaporator parameters

Trial	Temperature	RPM	Observations
1	45 °C	150	Thick film was formed
2	40 °C	150	Film was unevern
3	40 °C	100	Film cracked
4	40 °C	200	Thin and uniform film was formed

c. Selection of probe sonicator parameters

The results (Table S2) indicated that probe sonicator parameters showed a significant effect on particle size and drug EE. Hence, these parameters were selected for further optimization.

Table S2: Optimization of probe sonicator parameters

Trial	Sonication time (min)	Sonication Amplitude	Pulse time (on/off)	Particle size (nm)	EE (%)
1	5	40	4-2	209.9	59.1
2	10	20	10-2	219.6	52.02
3	5	20	10-2	135.7	50.71
4	10	40	4-2	219.1	45.01

S2.2 Confirmation of MCS polymer by FTIR and DSC analysis:

FTIR analysis was done to check the formation of MCS polymer, while DSC analysis was performed to check the thermostability of the polymer.

The chitosan mannosylation process involves the coupling of the aldehyde functional group of mannose to the amine functional group of chitosan using sodium triacetoxyborohydride as a reductive amination reagent according to the following reaction:

R-COH+R'-NH2 → R-CH-NH-R'

As a result, the only variation between chitosan and MCS functional groups is the existence of a N-H and CN bonds of secondary amine. However, any shift in the NH group will not be detected due to the existence of a lot of OH groups in chitosan and MCS. Figure S1 shows the FTIR spectra of mannose, chitosan, and MCS. The mannose spectrum shows multiple peaks related to sugar moieties and the chitosan spectrum shows a broad peak around 3430 cm-1 relating to the presence of amine and alcohol functional groups and a peak at around 1650 cm-1 is for N-H bending of the primary amine functional group of chitosan. For MCS polymer a peak at 1259 cm-1 is for C-N bond stretching, confirming the successful conjugation of mannose into chitosan [12].

The thermogram of mannose (Figure S2) showed an onset of peak at 132.94 °C with the maximum occurrence at 140.42 °C, and the chitosan (Figure S2) showed peak from 76.18 °C to 126.45 °C with maximum occurrence at 104 °C. While the thermogram of MCS polymer (Figure S2) shows two peaks at 85 °C and 105 °C which are of chitosan and mannose respectively. However, there is a little shift in the peak confirming the formation of a polymer. Since both the peaks are prominent chitosan and mannose show thermostability [12].

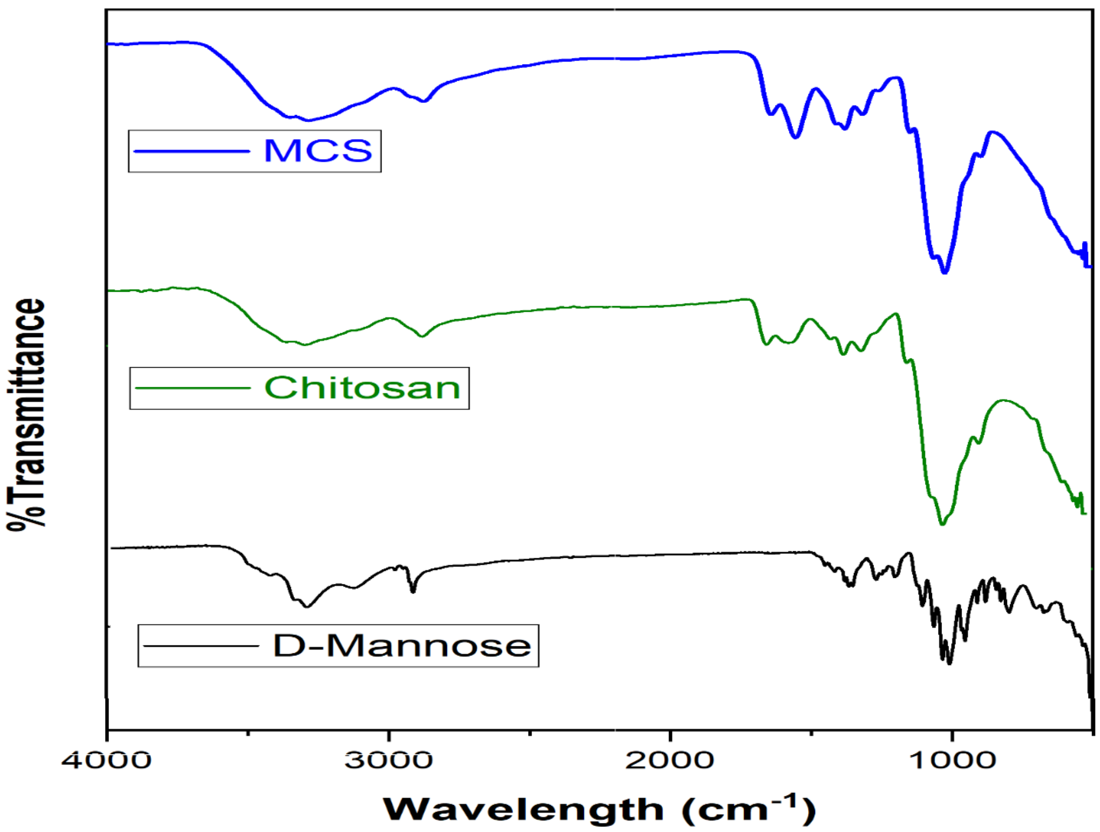

Figure S1: FTIR spectra of D-mannose, Chitosan, and MCS polymer

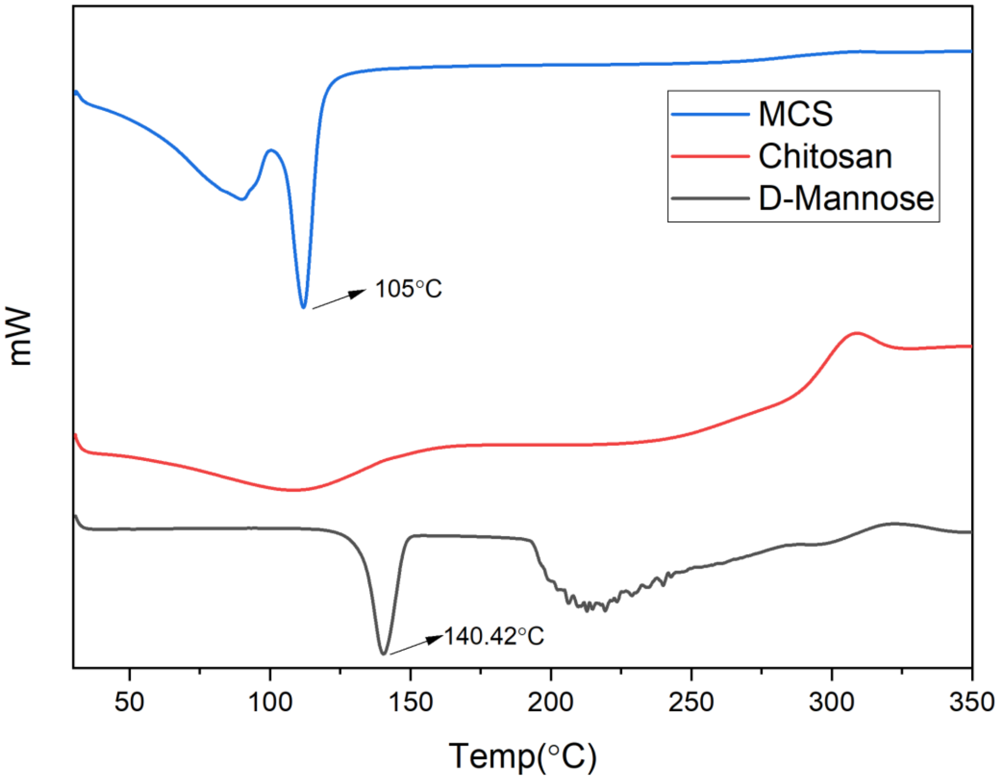

Figure S2: DSC thermogram of D-mannose, Chitosan, and MCS polymer
